# Surface Levels of CD20 Determine Anti-CD20 Antibodies Mediated Cell Death *In Vitro*

**DOI:** 10.1371/journal.pone.0111113

**Published:** 2014-11-03

**Authors:** Vijay Singh, Damodar Gupta, Rajesh Arora, Rajendra Prashad Tripathi, Alexandru Almasan, Roger M. Macklis

**Affiliations:** 1 Division of Radiation Biosciences, Institute of Nuclear Medicine & Allied Sciences, Brig SK Mazumdar Marg, Timarpur, Delhi, India; 2 Department of Radiation Oncology, Taussig Cancer Institute, Cleveland Clinic, Cleveland, Ohio, United States of America; 3 Department of Cancer Biology, Lerner Research Institute, Cleveland Clinic, Cleveland, Ohio, United States of America; University Paris Sud, France

## Abstract

**Background:**

The sensitivity of human Burkitt's lymphoma cells to rituximab (Rtx) and tositumomab (Tst) was assessed on cells expressing different levels of CD20 on surface. Cells that harbor low CD20 levels may resists against therapeutics response to CD20-specific antibodies. We postulated that, radiation-induced modulation of CD20 surface levels may play a crucial and central role in determining the relative efficacy of rituximab and tositumomab in treating Burkitt's lymphoma disease. Here, we examined the γ-radiation-induced CD20 expression in the Burkitt lymphoma cell line ‘Daudi’ and the relation of differential levels of CD20 with anti-CD20 mAbs mediated cell death.

**Methodology:**

In this study we examined kinetics of CD20 expression following sub lethal doses ofγ-radiation to Daudi cells and thereafter anti-CD20 mAbs (rituximab and tositumomab) were added in cell suspensions. The correlation of kinetics of CD20 expression and cells treated with anti-CD20 mAbs/or corresponding isotype Abs with special reference to changes in mitochondrial membrane potential and reactive oxygen species generation was also examined. Further, we also investigated the efficacy of anti-CD20 mAbs and possible induction of cell death in relation to levels of CD20 cell surface expression.

**Conclusion:**

This report provides evidence that CD20 expression can be induced by exposure of cells to γ-radiation. In addition, these findings demonstrated that the efficacy of anti-CD20 mAbs is dependent on the surface levels of CD20. Based on these findings, we hypothesized (i) irradiation just prior to immunotherapy may provide new treatment options even in aggressive B cell tumors, which are resistant to current therapies *in vivo* (ii) The efficacy of induction of apoptosis varies with type of monoclonal antibodies *in vitro*.

## Introduction

Over three decades, antibody cancer therapeutics have been established and used clinically in an effort to appreciate the potential of targeted therapy. In particular, the use of monoclonal antibodies have been shown with considerable achievement in the treatment of lymphoma and breast cancer [1].

CD20 a non-glycosylated transmembrane protein, exclusively expressed on B cells. It appears during the pre-B cell stage, however absent during the earlier or later stages of B cell differentiation such as antibody secreting plasma cells [2], [3]. CD20 has received extensive evaluation as an ideal target for immunotherapy and radio-immunotherapy, in part because of its ubiquitous expression, stable localization within the cell membraneof target cells (transformed B cells) [4]. Human CD20 has four membrane-spanning domains with a single extracellular loop and intracellular N-and C-terminal regions and forms a homo-oligomeric calcium ion channel complex [5]. Various monoclonal antibodies (mAbs) have been raised against CD20, which exerts various effects upon ligation and can be classified into type I and type II mAbs based on their ability to induce the reorganization of CD20 molecules into lipid rafts [6]. Type I antibodies, such as rituximab (Rtx) are distinguished by their ability to redistribute CD20 into lipid rafts and induce complement-dependent cytotoxicity (CDC) *in vivo* [6]. This activity appears to be directly linked to the translocation of CD20 and anti–CD20 mAbs complex into lipid rafts. Type II antibodies, such as tositumomab (Tst) do not considerably change CD20 distribution on ligation and relatively ineffective in CDC without the concomitant clustering *in vivo*. Intriguingly, they evoke far more homotypic adhesions (aggregations) and direct killing of target cells [7], [8], [9]. Both type I and II mAbs are capable of efficient antibody-dependent cellular phagocytosis (ADCP), antibody-dependent cellular cytotoxicity (ADCC) and can directly induce programmed cell death (PCD) [10], [11], [12]. It is now emerging that fine specificity differences exist in the epitope binding site of different anti-CD20 mAbs, formation of lipid rafts, and thereby initiation of intracellular signalling, may determine their biologicalefficacy *in vitro* and *in vivo* [13]. However, the acquired resistance in B-cell lymphomas following exposure to anti-CD20 mAbs may also be associated with reduced surface levels of CD20 and induce modest levels of cell death [14], [15], [16], [17], [18]. A range of signalling events are induced following ligation of CD20 with mAbs, *viz.* the activation of members of the src family of tyrosine kinases, elevation in intracellular Ca^2+^, phospholipase Cγ activation [19], [20], mitogen activated proteins kinase cascade [21], [22] and STAT3 down regulationof anti-apoptotic proteins like Bcl-X_L_, Bcl-2, [23], [24]. The earlier report suggests that the chimeric anti-CD20 mAb (Rtx) and cross-linking Fab'2 fragment, on B-cell chronic lymphocytic leukaemia cells (B-CLL) induce apoptosis through p^38^ MAP-kinase activation [21]. It has also been reported that the radiation and the type II anti-CD20 mAb (Tst) combine to evoke enhanced levels of cell death compared with either treatment alone through the MAPK signalling pathway downstream of ERK1/2 [22]. Radiation-induced changes in CD20 expression on B cells were evidenced first time in 1997 by Philippe et al [25]. Later on, Kunala et al have studied in more detail on various B lymphoblastoid cells types *viz*. Raji, Ramos, IM9 [26] and recently Gupta et al have investigated that low dose IR exposure to cells alters intracellular redox status, which determines expression of CD20 on malignant B cells [27]. The combination of ionizing radiation (IR) exposure and anti-CD20 mAbs in relation to therapeutic efficacy at surface levels of CD20 is poorly understood. Therefore, the ability to selectively control CD20 expression may be of great importance in enhancing therapeutic value and in optimizing anti-CD20 immunotherapy and radio-immunotherapy the modulation in CD20 expression may provide more binding sites for anti-CD20 mAbs and may play a major role in therapeutic response. Several investigators have demonstrated that mAbs which trigger direct PCD can stimulate the production of reactive oxygen species (ROS) and alters intra-cellular redox balance [28], [29], [30].

Improvement of antibody mediated cellular cytotoxicity or programmed cell death by modulation of CD20 surface level would also be an ideal approach. Since, ionizing radiation increases the surface levels of CD20 in treatment of Burkitt's lymphoma. We have under taken present investigation to compare the induction of cell death by Rtx and Tst mAbs and their relation with CD20 cell surface expression *in vitro*. Moreover, initiation of the signalling pathways following ligation of anti-CD20 mAbs and thereby leading to cell death is of considerable interest specifically in relation to surface levels of CD20 and therefore, we additionally expanded our investigation on mode of cell death *in vitro* following treatment of cells with Rtx and Tst mAbs. In current investigation, our data strongly suggests that type II antibody is strong inducer of cell death, which is mediated through p53 pathways *in vitro*.

## Results

### CD20 expression

**Figure 1:**
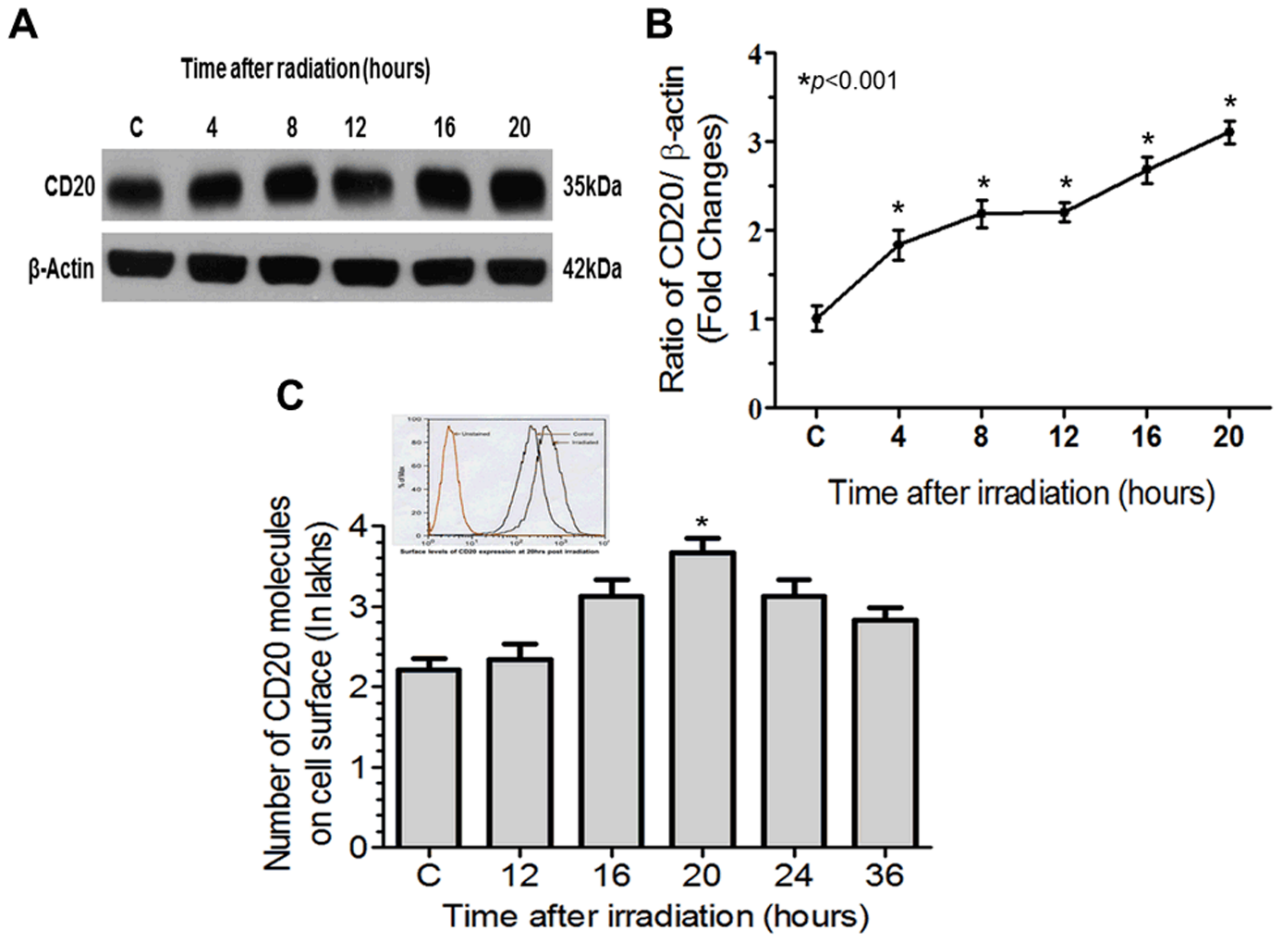
The kinetics of changes in CD20 expression on Daudi cells following exposure to 0.5 Gy γ-radiation. (A and B) Total CD20 levels: The changes in CD20 expression were measured using anti-CD20 antibodies. Results are expressed as ratio of levels of CD20 (CD20/β-Actin) with respect to 0 hr control. (C) CD20 expression on cell surface: The levels of CD20 at cell surface levels were calculated using quantiBRITE beads and expressed as numbers of CD20 molecules/cell. The index histogram is showing levels of CD20 at 20 hr (0.5Gy) with respect to 0 hr. Statistical analysis was done using Student's t-test (***p<0.001).

The kinetics of radiation-induced changes in total CD20 expression was measured at different time intervals as shown in Figure 1A. The increase in CD20 expression was found to be significantly higher at all-time intervals studied (4–20 hr) with respect to sham irradiated control (*p<0.001) (Figure 1B). However, maximum (2±0.4 fold) increase was observed 20 hr after irradiation.

The Increase of surface levels of CD20 was also found to be maximum at 20 hr (3.8±0.1 Lakh/cell) and remained higher till 36 hr and thereafter it reached to near sham irradiated control (2±0.2 Lakh/cell) with increase of time (Figure 1C).

### Changes in ROS

**Figure 2:**
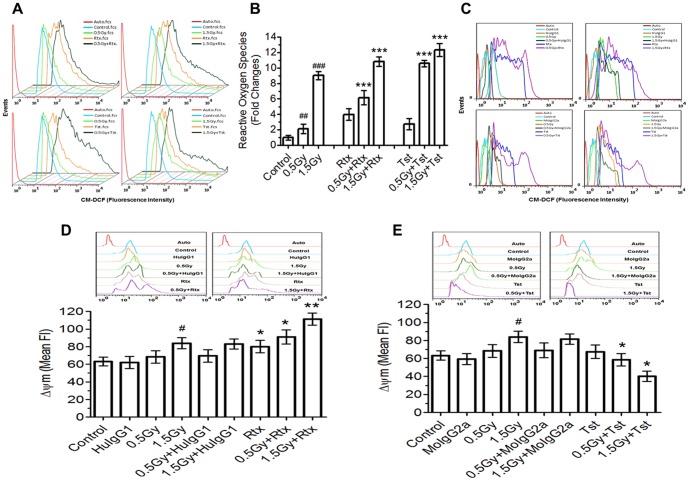
The changes in ROS and MMP (ΔΨm). (A, B and C) To measure changes ROS levels, fluorescence of CM-DCF was acquired at Ex λ of 488±10 nm and Em λ 517–527 nm and the results are expressed as the mean fluorescence ±SD of three independent experiments and statistical analysis was performed using ONE way ANOVA. Significant values are represented as; ***p<0.001 for Rtx *vs* 0.5Gy+Rtx or 1.5Gy+Rtx, ***p<0.001 for Tst *vs* 0.5Gy+Tst or 1.5Gy+Tst, ##*p*<0.01 for sham irradiated control *vs* 0.5Gy, ##*p*<0.001 for sham irradiated control *vs* 1.5Gy. Corresponding Isotype controls antibodies were used to measure changes in ROS levels. (D and E) The changes in ΔΨm were expressed from the mean fluorescence ±SD of three independent experiments and statistical analysis was performed using ONE way ANOVA. Significant values represents as; #p<0.05 for control *vs* 1.5Gy, *p<0.05 for control *vs* Rtx or 0.5Gy+RTX or 0.5Gy+Tst or 1.5Gy+Tst, **p<0.01 for control *vs* 1.5Gy+Rtx. Corresponding Isotypic controls were used to measure changes in ΔΨm levels [Human IgG1 (for Rtx) and Mouse IGG2a (Tst)].

The changes in intracellular ROS levels following various treatments *viz*. radiation (0.5Gy and 1.5Gy), Rtx and Tst and their combinations (IR+ anti-CD20) were measured as mentioned in Figure 2A, B and C. Cells exposed to IR (0.5Gy and 1.5Gy) showed significantly higher levels of ROS with respect to sham irradiated control (Figure 2B). However, 1.5Gy exposures to cells were found to be 7±0.35 fold higher with respect to 0.5Gy irradiated cells. Cells treated with Rtx and Tst also showed increased the levels of ROS by 3.9±0.3 and 2.7±0.29 folds with respect to sham irradiated control respectively (Figure 2A, B). Cells exposed to low dose of radiation (0.5Gy) were further treated (at +20 hr) with Rtx showed 5.1±0.62 and 3.9±0.35 fold increase in ROS levels with respect to sham irradiated control and 0.5Gy respectively (Figure 2A, B). However, cells exposed to 0.5Gy (at +20 hr) +Tst showed 9.5±0.4 and 7±0.7 fold with respect to sham irradiated control and 0.5Gy respectively. Moreover, cells exposed to 0.5Gy (at +20 hr)+Tst showed 7.2±0.6 fold increase ROS as compare to Tst alone. Furthermore, 1.5Gy (at +20 hr) +Rtx and 1.5Gy (at +20 hr) +Tst showed significantly higher levels of ROS with respect to 1.5Gy radiation as well as 1.5Gy (at +20 hr)+Tst also showed significant difference respect to 1.5Gy (at + 20 hr) +Rtx (Figure 2A, B). The changes in generation of ROS were found to be insignificant when cells treated with corresponding isotypic antibody (Figure 2C).

### Changes in mitochondrial membrane potential (MMP; Δψm)

The change in mitochondrial membrane potential is indicator of cellular responses in terms of treatment(s). Cells exposed to γ- radiation, treated with mAbs and/or combination of radiation with mAbs showed significant changes in ΔΨm as compared to control group (Figure 2D, E). Cells treated with Rtx alone or in combination of radiation and mAbs showed significant increase in ΔmΨ as compared to sham irradiated control (*p*<0.05; Figure 2D), whereas in case of cells exposed to radiation (0.5Gy or 1.5Gy) followed by treatment with Tst showed significant loss of ΔΨm with respect to Tst alone and sham irradiated control (*p*<0.05; Figure 2E). However cells exposed to 0.5Gy or 1.5Gy and treated with isotype control antibodies alone or in combination showed no significant changes in ΔΨm with respect to sham irradiated control or corresponding irradiated groups (Figure 2D, E).

### Induction of cell death and cell cycle distribution

**Figure 3:**
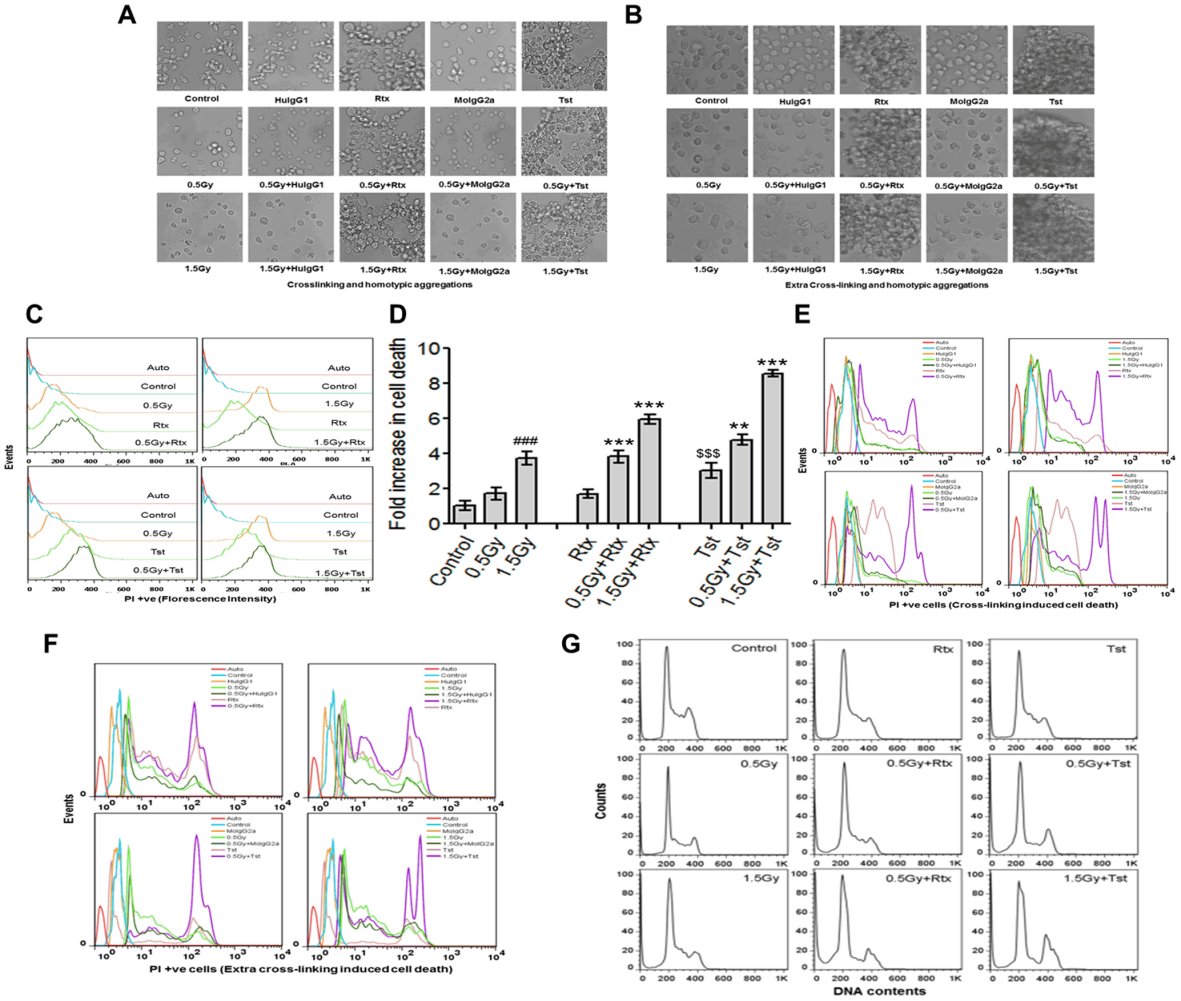
Cross-linking or homotypic adhesions (aggregations) and cell death. (A–B) The binding of Fab regions of Anti-CD20 mAbs (Rtx and Tst) on CD20 and Fc region binding to FcγRIIB1 thereby induction of cross-linking or homotypic adhesions (aggregations) as well as extra cross-linking induced by corresponding secondary antibodies were observed microscopically (20x). Isotype control antibodies were taken separately to measure non-specific cross-linking. (C, D and E) Anti-CD20 mAbs induced cell death was measured using PI uptake by flowcytometrically. PI is membrane impermeable, generally excluded from viable cells and therefore, commonly used for identifying alterations in biological membrane and thereby death of cells in a population. The fold induction cell death was measured from mean fluorescence ±SD and statistical analysis was performed using ONE way ANOVA. Significant values represented as; ***p<0.001 for Rtx *vs* 0.5Gy+Rtx and 1.5Gy+Rtx. ***p<0.001 for Tst *vs* 1.5Gy+Tst, **p<0.01 Tst *vs* 0.5Gy+Tst. Further, ###*p*<0.001 for sham irradiated control *vs* 1.5Gy, and $$$*p*<0.001 for sham irradiated control *vs* Tst. Isotype control antibodies were taken separately to measure non-specific induction of cell death. (F) Induction of cell death by extra cross-linking using secondary antibodies: Cells expressing different levels of CD20 and treated with monoclonal anti-CD20 antibodies or isotypes (separately) were further incubated with corresponding secondary antibodies. PI uptake was also measured flowcytometrically to measure extra crosslinking induced cell death. (G) Cell cycle analysis: For cell cycle analysis, binding of PI with DNA were used as a marker of DNA content which depicts phase of cells in cell cycle.

The Fab regions of anti-CD20 mAbs bind to CD20 and Fc region bind to FcγRIIB1. They were exclusively expressed on B cells and induce Cross-linking and homotypic adhesions (aggregations). As shown in Figure 3A large aggregates of cells were observed following treatment of cells with Tst alone as compared to Rtx alone. Cells treated with isotype antibodies showed no cross-linking or aggregation. However, extra cross-linking was induced by using corresponding secondary antibodies such as anti-mouse for Tst and anti-human for Rtx (Figure 3B). An apparent cell death due to cross-linking or extra cross-linking was measured flowcytometrically using PI uptake (Figure 3C to F). As shown in Figure 3C and 3D cells treated with Rtx alone or in combination (0.5Gy+Rtx and 1.5Gy+Rtx, 20 hr post irradiation) showed 2.5±0.4 and 4.5±0.2 increase fold cell death respectively as compared to Rtx alone (***p<0.001). Whereas, cells treated with Tst alone or their combination 0.5Gy+Tst (20 hr post irradiation) showed 1.5±0.2 fold increase in cell death (**p<0.05) and 1.5Gy+Tst (20 hr post irradiation) showed 5.7±0.3 fold increase in cell death (***p<0.001) compare to Tst alone (Figure 3C, D). Moreover, cells treated with isotype antibodies showed no cell death (Figure 3E). Furthermore, cells treated with corresponding secondary antibody after treatment of Rtx or Tst also showed high induction of cell death compared to Rtx or Tst treated alone (Figure 3F).

The changes in cell cycle phase distribution and apoptosis following radiation and treatment with anti-CD20 mAbs are shown in Figure 3G and Table 1. With respect to control all treatments (radiation, mAbs and/or combinations) showed significant changes in cell cycle phase distribution. Cells exposed to 0.5Gy gamma radiation and further treated with Rtx and Tst showed significant increased G2/M block with respect to sham irradiated control. Moreover, the decrease in DNA synthesis phase in case of combinations (IR + mAbs) found to be significantly less with respect to corresponding controls (0.5Gy, 1.5Gy and mAbs).

**Table 1:** Effects of anti-CD20 mAbs on the cell cycle phases.

S.No.	Sample	% of cells in G1	% of cells in S	% of cells in G2/M	% Cell death
1	Control	43.70±6.5	14.80±2.1	34.77±4.4	6.73±0.6
4	0.5Gy	38.10±5.8	9.60±1.2	42.70±3.2	9.60±1.0
3	1.5Gy	40.20±4.3	6.70±1.4	41.60±4.7	11.50±0.9
2	Rtx	34.50±2.4	11.26±1.9	45.44±4.9	8.80±0.7
5	0.5Gy+Rtx	40.02±3.2	6.98±1.9	42.90±4.9	10.10±0.4
6	1.5Gy+Rtx	38.75±6.1	2.75±1.6	43.40±5.4	15.10±1.0
3	Tst	40.55±5.2	6.52±1.8	43.08±3.1	9.90±1.1
8	0.5Gy+Tst	32.40±3.5	3.63±1.9	48.40±3.5	15.57±1.3
9	1.5Gy+Tst	25.60±7.3	1.38±1.6	53.90±4.5	19.12±1.04

Rtx  =  Rituximab, Tst  =  Tositumomab.

### Alkaline comet assay

**Figure 4:**
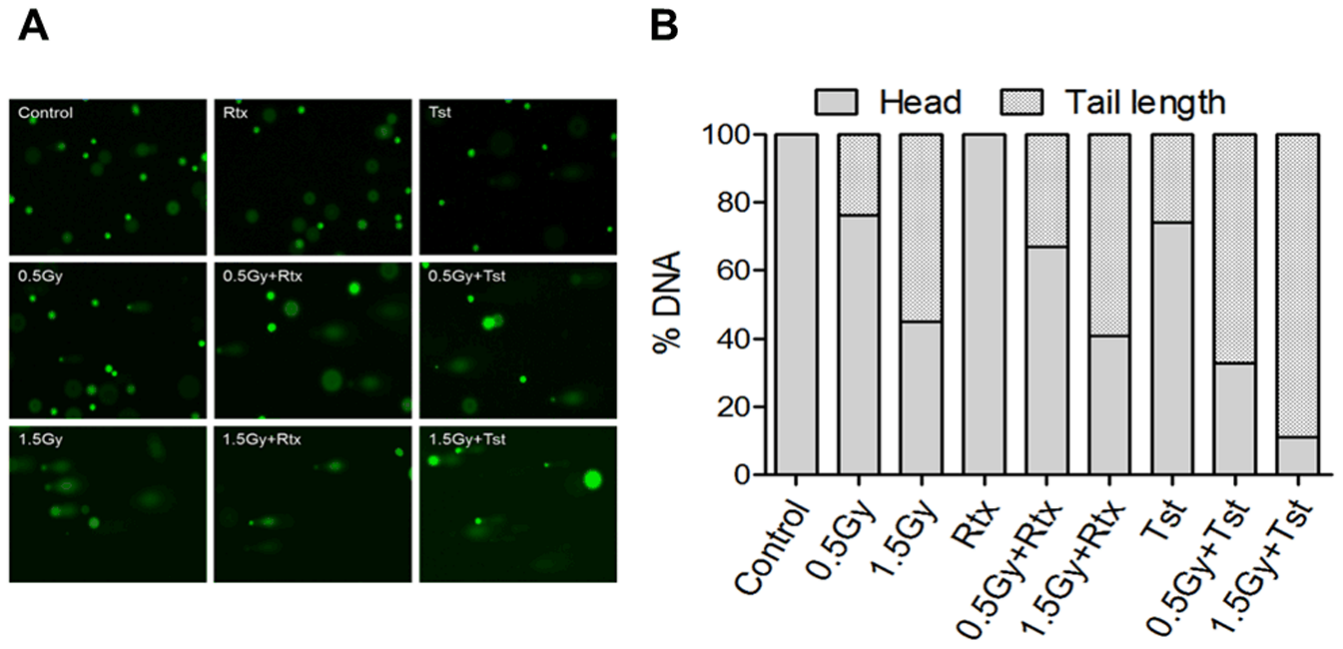
Comet assay. (A) Samples were subjected to alkaline comet assay to monitor DNA damage stained with SYBR Green I and Exλ 488 nm, Emλ 520 nm. Images were capture using Olympus BX60 florescence microscope at 20x. (B) Results are expressed as % DNA content in head and tail region.

The genotoxic stress was assessed using alkaline comet assay as shown in Figure 4 A and 4B. Cells exposed to γ-radiation (0.5Gy and 1.5Gy) showed significant increase of DNA damage. The radiation induced damage to DNA was found to be dose dependent. Cells exposed to 0.5Gy or 1.5Gy separately, and further treated with Tst at 20 hr post irradiation interval showed higher induction of DNA damage as compare to corresponding irradiation dose alone or Tst alone (Figure 4A, B). Whereas, cells treated with Rtx alone or combination with radiation (0.5Gy+Rtx and 1.5+Rtx, 20 hr post irradiation) did not show significant DNA damage as compared to sham irradiated control. The greatest frequency of induction of DNA damage was observed in case of 1.5Gy+Tst (20 hr post irradiation) respect to IR or mAbs.

### Immunobloting

**Figure 5:**
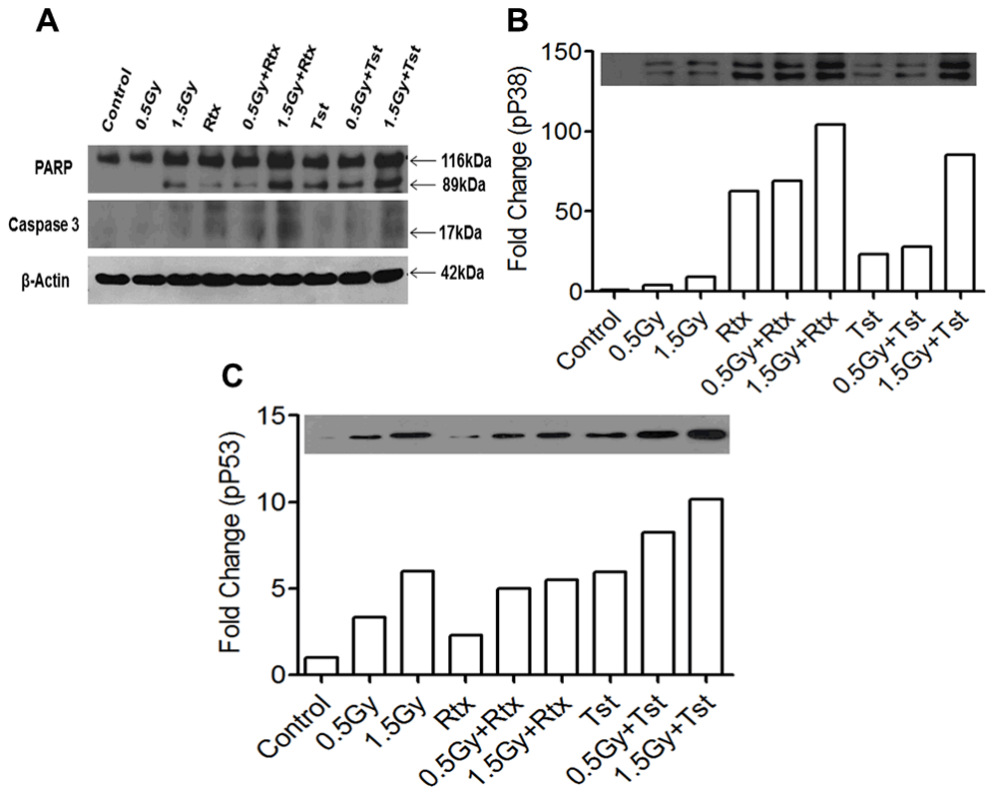
Cell death induced by monoclonal anti-CD20 Abs: Cell death was measured in cells expressing different levels of CD20 followed by treatment with monoclonal anti CD20 Antibodies. (A) For measurement of apoptosis cells were lysed and equal quantity of proteins loaded and resolved in a 12% Tris–HCl gel, followed by transfer onto nitrocellulose membrane, and immunoblotted for (cleavage of both PARP and caspase 3) (B) p38 at site Thr180/Tyr 182 also were immunoblotted as described in methods section,(C) Phosphorylation of p53 at site ser15 and β-actin were used as loading control.

Activation of caspase-3 and cleavage of PARP were assessed following various treatments as a marker of apoptosis. As shown in Figure 5A exposure of cells with radiation Rtx alone or combination of radiation with Rtx showed activation of caspase-3 and cleavage of PARP. However combination of radiation (0.5Gy/1.5Gy) with Rtx (20 hr post irradiation) showed significantly higher levels of active caspase-3 and cleaved PARP with respect to Rtx alone or sham irradiated control. Treatment of cells with Tst alone or combinations of radiation with Tst (0.5Gy+Tst and 1.5Gy+Tst) have shown significant increase of PARP cleavage (Figure 5A). To further elucidate the mode of cell death induced by anti-CD20 mAbs, changes in phosphorylation of p38 (Tyr180/Trp182) and p53 (ser15) were measured as shown in Figure 5B and C. The potential effect of p38 on cell death induced by anti-CD20 mAbs with and without radiation was assessed as phosphorylation of p38 at Tyr180/Trp182. Control, 0.5Gy and 1.5Gy groups showed negligible activation/phosphorylation of p38 (Figure 5B). Whereas, cells treated anti-CD20 mAbs (Rtx and Tst) with or without radiation showed significant activation of p38 with respect to control or mAbs or IR alone (Figure 5B). With respect to 0.5Gy alone, 0.5Gy (20 hr post irradiation) +Rtx and 0.5Gy (20 hr post irradiation) +Tst showed 6±0.5 and 2.5±0.5 folds activation. Moreover, corresponding to 1.5Gy, 1.5Gy+Rtx and 1.5Gy+Tst found to be 11±0.5 and 8.5±0.5 folds activation of p38 (Figure 5B). The level of phosphorylation of p53 was found to be increased with radiation dose. However, in case of combination of radiation with Tst, it was found to be significantly higher with respect to radiation alone (Figure 3C).

## Discussion

In the present investigation, we have evaluated differential responses of anti-CD20 antibodies (Rtx and/or Tst) on Daudi cells bearing different levels of CD20. We identified the induction of cell death induced by mAbs is associated with the CD20 cell surface expression. Cells exposed to sub-lethal dose of γ-radiation (0.5Gy) significantly increased CD20 expression in time dependent manner (Figure 1A, B, and C). The results are also correlated with our previous findings related to redox regulation of CD20 expression, where we studied the expression of CD20 is upto a threshold and the increase of surface levels of CD20 is independent of radiation dose and time [27]. Based on hypothesis of increase in surface levels of CD20 may be beneficial during immunotherapy/radio-immunotherapy to achieve certain clinical benefits of the targeted mAbs by assuming more targets-more association of mAbs, we studied possible changes in intra-cellular signalling in relation to ligation of mAbs with CD20. Moreover cells expressing different levels of CD20 (sham irradiated control and 0.5/1.5Gy irradiated, +20 hr) were treated with anti-CD20 mAbs and changes in levels of ROS, ΔΨm and initiation of cell death were measured to understand cellular responses following ligation of anti-CD20 mAbs and its relation with cell surface levels of CD20. Ionizing radiation is known to induce generation of reactive oxygen species (ROS) and alters ΔΨm, which may lead to alterations in cellular redox balance and/or cell death [27], [31]. In the present investigation cells exposed to radiation showed increases in ROS levels with respect to control, which is found to be radiation dose dependent (Figure 2A, B). Micro-level changes (due to 0.5Gy) in generation of ROS may alter cellular redox balance/ROS mediated signalling, which may be responsible for increased CD20 expression both at total cellular and cell surface levels as also reported earlier [27]. Cells with increased expression of CD20 and thereafter treated with anti CD20 mAbs (Rtx or Tst) showed significant increase in both ROS generation and ΔΨm, which could be due to increase of interaction of anti-CD mAbs, as cells possess increase surface levels of CD20 (Figure 2A to E). The interaction/association of anti-CD20 mAbs is known to induce generation of ROS and thereby alters intracellular physiology [32]. The fold changes in both ROS and ΔΨm found to be significantly higher in combination (IR + mAbs) with respect to corresponding controls and also suggests that the levels of cell surface CD20 plays major role in anti-CD20 mAbs mediated cell death.

It is well known that anti-CD20 mAbs can activate classic Fc-dependent mechanisms such as ADCC, ADCP, CDC under *in vivo* condition [6], [10], [11]. However, certain anti-CD20 mAbs can eliminate B cells by triggering intracellular signalling on ligation with antigen and directly induce programmed cell death (PCD) *via* cross-linking and homotypic adhesions (aggregations) [12]. In this report we found that the cross-linking and homotypic adhesions (aggregations) were higher in combination (IR + mAbs) as compared to normal CD20 expression (Figure 3A). Cragg et al distinguished that the rituximab-like mAbs translocate CD20 into lipid rafts and promote complement-mediated lysis whereas Tst-like mAbs do not translocate CD20 into conventional lipid rafts, but encourage programmed cell death [6]. Moreover, The cross-linking of chimeric anti-CD20 mAbs is known to activate, members of the src family of tyrosine kinases and thereby induces cell death, [19], [20], [21], [22], [23], [24].

Earlier it was also reported that the cross-linking Fab'2 fragment of chimeric anti-CD20 mAb rituximab induce apoptosis and the influence of complement activation and ADCC was negligible [21]. In this report, we have shown that *in vitro* induction of cell death *via* cross-linking and homotypic adhesions of Rtx or Tst as well as extra cross-linking induced by using corresponding secondary antibodies (Figure 3A, B). Cell death induced by Rtx on ligation with CD20 found to be *via* activation of p38 MAP-kinase (Figure 5B), whereas Tst was found to be potent inducer of p53 pathway (Figure 5C) and results are corroborated with DNA damage as measured by comet assay and the damage to DNA was found to be significantly higher with respect to cells treated with Rtx alone. These findings are also corroborated with findings of Deans et al [20], Hofmeister et al [19], Pedersen et al [21] and Ivanov et al [22].

Cells expressing higher levels of CD20 and further treated with either Rtx or Tst have shown better induction of cell death with respect to control cells (sham irradiated) treated with anti-CD20 mAbs (Rtx or Tst). In addition to the recent progress in understanding of how type I and II mAbs might engage CD20 differently, as detailed above, our findings suggests that there is unique mode of cell death in response to type II mAbs and it varies with differential levels of CD20. For many years it has been appreciated that Rtx and other type I anti-CD20 mAbs can mediate direct cell killing particularly when hyper–cross-linked with CD20 or by the use of mAbs oligomer conjugates [33], [34]. This process is somewhat controversial but often bears hallmarks of classical apoptosis and appears largely caspase-dependent; however, it is not clear whether anti-CD20 mAbs (Rtx) cross-linking *in vivo*, for example by FcγR, could ever deliver this form of killing. In contrast to type I (Rtx), Type II CD20 mAb or Tst are able to induce direct cell death upon binding to CD20 as caspase-independent manner and found to be potent inducer of p53 pathway and DNA damage (Figure 5A, B, and C).

**Figure 6:**
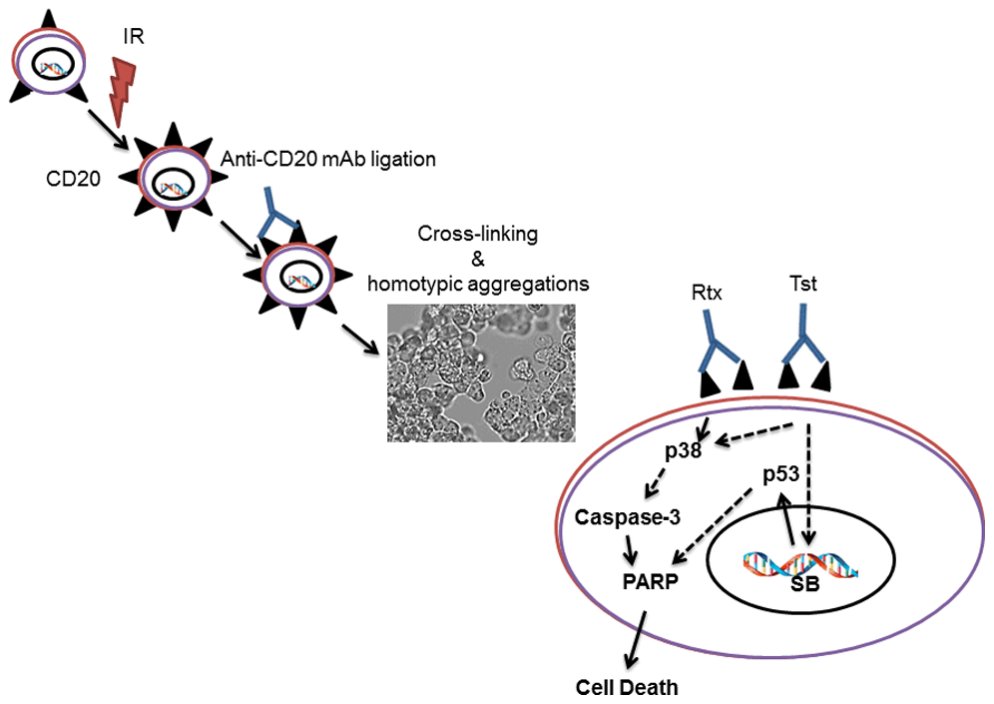
Anti-CD20 mAbs-induced programed cell death. Schematic diagram illustrating the kinetics of changes in CD20 expression followed 0.5Gy γ-radiation and sequence of events in the proposed possible cell death pathway evoked by type I anti-CD20 mAbs (Rtx) and type I anti-CD20 mAbs (Tst). Anti-CD20 mAbs ligation results in cross-linking and homotypic adhesions followed by generation of ROS and create genotoxic stress which ultimately culminates in apoptotic and non-apoptotic cell death. *SB =  Strand Break.

Present investigations suggest that increase in cell surface CD20 expression is associated with increase cell death induced by anti-CD20 mAbs as schematic representation in Figure 6. In conclusion, this report provides evidence that CD20 expression can be induced by low dose γ-radiation. Therefore, use of irradiation just prior to immunotherapy may be beneficial for eradication of B cell malignancy. Further, it has been described that *in vitro* cytotoxic effects of Rtx, is mediated by a caspase-dependent processes that involves ROS generation, and alteration in ΔΨm, whereas in case of Tst, cell death occurred by a caspase-independent/non-apoptotic cell death process that involve ROS generation and DNA damage/p53 activation. These results may be useful to establish a theoretical basis to improve the efficacy of immunotherapy/radio-immunotherapy in case of cells expressing low surface levels of CD20.

## Materials and Methods

RPMI-1640, Penicillin G potassium salt, streptomycin, N-2-Hydroxyethyl piperazine N-2-Ethane sulphonic acid sodium (HEPES sodium), Dimethyl sulfoxide (DMSO), sodium pyruvate, Bradford reagent, Protease and Phosphatase inhibitor Cocktail were obtained from Sigma Aldrich Chemicals (St. Louis, MO, USA). Fetal bovine serum (FBS), 5-(and 6)-chloromethyl-2′7′-dichlorodihydrofluorescein diacetate acetyl ester (CM-H_2_DCFDA), 3, 3′-dihexyloxacarbocyanine iodide (DiOC_6_) (3), Propidium Iodide (PI) and dihydroethidium (DHE) were obtained from was obtained from Invitrogen (USA). Anti-CD20 chimeric antibodies, such as rituximab (Rtx) were obtained from Genentech (Genentech, Inc., South San Francisco, CA), and tositumomab (Tst) from Corixa (Corixa Corporation Seattle, WA). QuantiBRITE beads and anti-CD20-PEwas obtained from BD bioscience (USA). Human IgG1 (HuIgG1) and Mouse IgG2a (MoIgG2a) were obtained from Sigma Aldrich Chemicals (St. Louis, MO, USA). All other chemical used were of AR grade and obtained from local manufacturers from SRL and Himedia India. Cell culture and biochemical purpose related plastic wares were obtained from BD bioscience (USA), Corning (USA) and Tarsons, INDIA.

### Cell culture

Daudi cells was obtained from the American type culture collection, (USA) and maintained in RPMI 1640 supplemented with 10% fetal bovine serum, 10 mM HEPES, 50units/ml penicillin, 50 µg/ml streptomycin, and 1% non-essential amino acids at 37°C in a humid atmosphere with 95% air and 5% CO_2_.

### Irradiation and treatment of cells

Exponentially growing cells were exposed with γ-radiation, 0.5Gy and 1.5Gy separately using ^6^°Co-teletherapy unit (Bhabhatron-II Telecobalt unit; obtained from Bhabha Atomic Research Center (BARC), Mumbai, India) at a dose rate of 1.67Gy/min. Control groups were treated similarly except for irradiation. After irradiation cells were incubated at 37°C in a humid atmosphere with 95% air & 5% CO_2_ for further experiments.

Ionizing radiation (IR) induced changes in expression of CD20 was measured at different time intervals. Cells exposed to low doses of IR were further treated with anit-CD20 mAbs (5 µg/ml, Tst and Rtx respectively for all experiments) to measure their responses in combination as well as HuIgG1 (for Rtx) and MoIgG2a (for Tst) used to ensure non-specific induction of cell death induced by anti-CD20 mAbs. Individual sham irradiated controls and antibodies alone were taken separately.

### CD20 expression

The levels of changes in expression of total CD20 were measured using western blotting techniques (described later). The changes in levels of CD20 at the surface of cells were measured flowcytometrically using anti-CD20 antibodies conjugated with PE at an Ex λ 488 nm and Emλ 578 nm. The fluorescence was also acquired using quantiBRITE beads and numbers of CD20 at the surface of CD20 were calculated from standard curve obtained from fluorescence of beads.

### Reactive Oxygen Species generation and changes mitochondrial membrane potential

The intracellular reactive oxygen species (ROS) generation was determined by flowcytometry using CM-H_2_DCFDA [27], [35], [36]. Briefly, cells were harvested following different treatments and incubated with CM-H_2_DCFDA (20 µM) to determine free radical-mediated oxidation of dye at room temperature in dark condition. The fluorescence of the oxidized probe was acquired by FACS-Caliber (Becton, Dickinson, USA) from 20,000 cells at an Ex λ488 nm and Em λ 517-527 nm. The mean fluorescence was measured using FlowJo 7.6.5 (Becton, Dickinson, USA).

The mitochondrial membrane potential (ΔΨm) was measured as described by Gupta *et al* [27]. Briefly, cells (5×10^5^ per measurement) were stained with DiOC_6_ (3), 40 nM for 15 min at 37°C and thereafter fluorescence of DiOC6 (3) was acquired (Ex λ 488±10 nm and Em λ 530±10 nm) flowcytometrically. Nonspecific uptake of DiOC_6_ (3) was measured by treating cells with gramicidin (20 µg/ml for 30 min at 37°C), to abolish the membrane potential of stained cells. The data were analysed, using FlowJo7.6.5, and results are expressed as mean fluorescence ±SD.

### Cell death and Cell cycle phase distribution

The binding of Fab'2 domain of anti-CD20 mAbs with CD20 and Fc domain with FcγRIIB1 (exclusively expressed on B cells) is known to initiate cross-linking or homotypic adhesions (aggregations) as well as extra cross-linking using anti-human IgG (for Rtx) and anti-mouse IgG (Tst) secondary antibodies was observedmicroscopically(20x). Anti-CD20 mAbs induced cell death was measured as PI uptake by flowcytometrically. PI is membrane impermeable dye, generally excluded from viable cells and therefore, commonly used for identifying dead cells in a population. Briefly, cells were harvested and incubated with PI (5 µg/ml) at room temperature in the dark to determine death cells. The results are expressed as fold induction cell death from mean fluorescence ± SD of three independent experiments.

The changes in cell cycle phase distribution were measured flowcytometrically by using PI as previously described by Adhikari et al [27], [35]. Briefly, cells (1×10^6^cells/sample) were harvested and fixed in 70% ice cold ethanol. For cell cycle studies fixed cells were centrifuged (1500 rpm, 10 min at room temperature) and pellets were washed with phosphate buffer saline (pH 7.4) twice to remove traces of ethanol and thereafter treated with 200 µg/ml RNaseA at 37°C for 30 min. The cells were stained with PI (50 µg/ml) and % DNA content were measured using fluorescence-activated cell sorting (FACS) in a BD SLR II flow cytometer (BD Bioscience, USA) at Ex λ 488±10 nm and Emλ 617±10 nm. The Acquired results were analysed using FlowJo 7.6.5.

### Single cell gel electrophoresis

The damage to DNA was measured using single cell gel electrophoresis (comet assay) as previously described [35], [37], [38]. In brief, single cell suspensions were prepared andsuspended in low-melting point agarose (0.8%) in phosphate buffer saline (pH 7.4). The mixture of cells in LM agarose was gently layered on pre-coated slides (agarose 1% in Milli Q HPLC grade water) and allowed to form gel at 4°C. After polymerization of gel slides were merged in for lysis of cells in lysis buffer (2.5M NaCl, 100 mM EDTA, 10 mM Tris base, 1% Triton X-100, 1% N-Lorylsarcosine, pH 10), washed with ice cold MilliQ water and there after run at 4°C at 1V/cm for 30 min in running buffer (200 mM NaOH, 500 mM EDTA, pH 13.1). After electrophoresis, slides were washed with ice cold MilliQ water, dehydrated with 70% ice cold ethanol (7∶3; ethanol: water v/v) and thereafter dried in dark. The DNA in gel was stained with SYBR Green I and images were acquired using Olympus BX60 florescence microscope (Olympus optical Co, Tokyo, Japan), Ex λ 488 nm and Emλ 520 nmand the results are expressed as the mean ±SD

### Western blotting

To study the induction of cell death, expression and cleavage of various apoptotic marker proteins were measured as described by Gupta et al [27], [38], [39]. Briefly cell lysates were prepared in RIPA lysis buffer (50 mM Tris-HCl pH 7.4, 150 mM NaCl, 0.5% Sodium deoxycholate, 0.1% SDS, 1% Nonidat-P40, 5 mM EDTA, 1 mM EGTA, 1 mM PMSF, protease and phosphatase inhibitor cocktails) and protein quantity was measured using Bradford method [40]. Equal quantity of proteins (60 µg/sample) of different samples were run and resolved on denaturating 12% bis-acrylamide gel followed by transfer on to a nitrocellulose membrane. The membranes were blocked in blocking buffer (3% BSA prepared in tris buffer saline with 0.1% Tween-20) for 4 hrs followed by overnight incubation with desired primary antibody *viz.* anti-cleaved PARP (Ser214); 1∶2000, anti-Phospho-P53 (pSer15); 1∶1500, anti-Phospho-P38 (Try190/Trp182); 1∶1000, active caspase-3; 1∶1000 and anti CD20; 1∶2000. After incubation, membrane were washed and incubated with horseradish peroxidase-conjugated secondary antibodies (Sigma, St. Louis, MO, USA) for 4 hr at room temperature. The protein bands were visualized by using enhanced ECL chemiluminescence Pico. β – Actin was used to ensure equal loading of proteins.

### Data analyses and statistical evaluations

Assays were set up in triplicates and the results were expressed as the mean ±SD. Changes in ROS and ΔΨm were analysed by student's t-test, whereas for multiple the combination groups, one way ANOVA with Newman-Keuls Multiple Comparison to test the significance among the groups using the Graph pad Prism 5 (Graph Pad Software, San Diego, CA, USA). Optimas 5.0 comet assay module was used to analyse comet tail length and % DNA content in head and tail region. For the graphical representation of the data, y- axis error bars representing ±SD are depicted and *p* values were shown at different levels of significance (*p<0.05, **p<0.01, ***p<0.001).
